# CANINE COGNITION ASSESSMENTS IN THE TEST OF RAPAMYCIN IN AGING DOGS (TRIAD)

**DOI:** 10.1093/geroni/igae098.3639

**Published:** 2024-12-31

**Authors:** Sydney Holland, Anna Ferguson, Kate Creevy, Rozalyn Anderson, May Reed

**Affiliations:** Texas A&M University College of Veterinary Medicine and Biomedical Sciences, College Station, Texas, United States; University of Washington School of Medicine, Seattle, Washington, United States; Texas A&M University College of Veterinary Medicine and Biomedical Sciences, College Station, Texas, United States; University of Wisconsin-Madison, Madison, Wisconsin, United States; University of Washington School of Medicine, University of Washington, Washington, United States

## Abstract

The primary objective of the Dog Aging Project’s Test of Rapamycin in Aging Dogs (TRIAD), is to assess whether rapamycin treatment extends lifespan in healthy, middle-aged, medium-to-large companion dogs. A secondary objective is to determine whether rapamycin impacts cognitive function in aging dogs. This placebo-controlled, double-blinded, randomized clinical trial is underway; however, baseline data allows us to investigate relationships between demographic factors and cognitive function. Dogs who met medical records eligibility criteria for TRIAD were assessed during an in-person examination by veterinary neurologists. Baseline cognitive functional assessment involved two validated clinical assessments: the Sustained Gaze test (possible scores 1 to 60 seconds; higher scores indicate greater attention span), and the CAnine DEmentia Scale (CADES) (possible scores of 0-85; higher scores indicate greater impairment). Thirty-five dogs were examined for inclusion in TRIAD, including baseline assessment of cognitive function. The median age of evaluated dogs was 9.2 years (8.1-10.2) and the median weight was 27.7 kg (24.5-30.2). The median CADES score was 1.0 (0.0-4.0) and median Sustained Gaze score was 9.0 (5.3-14.0). Post hoc analysis did not identify significant correlations between age, weight, sex, or breed and scores for either cognitive test. CADES and Sustained Gaze tests do not reveal an influence of demographic factors on cognition at middle-age in dogs being assessed for enrollment in TRIAD. Accordingly, the TRIAD clinical trial will enable robust assessment of rapamycin’s impact on age-related cognitive decline.

